# Castleman’s disease and sarcoidosis, a rare association resulting in a “mixed” response: a case report

**DOI:** 10.1186/s13256-015-0517-8

**Published:** 2015-02-28

**Authors:** Anwarullah Mohammed, Filip Janku, Ming Qi, Razelle Kurzrock

**Affiliations:** 1 Department of Cardiology, Texas Heart Institute at St. Luke’s Episcopal Hospital, 6720 Bertner Avenue, Houston, TX 77030 USA; 2 Department of Investigational Cancer Therapeutics, The University of Texas MD Anderson Cancer Center, 1515 Holcombe Boulevard, Unit Number: 0455, Houston, TX 77030 USA; 3 Janssen R&D LLC, 1400 McKean Rd, Spring House (Ambler), PA 19002 USA; 4 UC San Diego Moores Cancer Center, 3855 Health Sciences Drive, MC #0658, La Jolla, CA 92093-0658 USA

**Keywords:** Interleukin 6, Mixed response, Multicentric Castleman’s disease, Siltuximab

## Abstract

**Introduction:**

Multicentric Castleman’s disease is a rare lymphoproliferative disorder whose hallmark is atypical lymph node hyperplasia. Symptoms can include fever, splenomegaly, and abnormal blood cell counts. High levels of interleukin 6 (IL-6) are observed frequently in this disorder and are believed to drive the disease. Recently, therapies that target interleukin-6 or its receptor have been shown to be effective in Castleman’s disease.

**Case presentation:**

We report the case of a 76-year-old Caucasian man with aggressive biopsy-proven Castleman’s disease who experienced pulmonary and lymph node involvement, as well as fever and weight loss. He was treated with siltuximab, a chimeric anti-interleukin-6 antibody. After 5 months, fluorodeoxyglucose positron emission tomography computed tomography scans showed marked improvement in his lungs, but worsening mediastinal disease, consistent with a mixed response. Biopsy of the mediastinal disease revealed lymphoplasmacytic infiltrate with non-caseating, ill-defined granulomas and scarring consistent with sarcoidosis. Prednisone 50mg by mouth daily was started, which was tapered to 2.0-5.0mg daily. Siltuximab was continued. A subsequent fluorodeoxyglucose positron emission tomography computed tomography scan showed near-complete resolution of lung and mediastinal disease, now ongoing for 3.5+ years without serious adverse events.

**Conclusions:**

Lymphomas have previously been reported to coexist with sarcoidosis, albeit rarely, but there has been only a single previous case of this type with Castleman’s disease. Of importance, early recognition of the presence of sarcoidosis in our patient prevented discontinuation of siltuximab therapy due to “progression”. Our experience may also have broader implications in that it suggests that etiology of “mixed responses” should be confirmed by performing biopsies on the progressive tumor.

## Introduction

Castleman’s disease is a rare lymphoproliferative disorder characterized by atypical lymph node hyperplasia that can involve any group of lymph nodes [1]. The etiology of the disease remains unclear, but recently it has been found to be increasingly associated with infection with human immunodeficiency virus (HIV) and human herpesvirus 8 (HHV-8), the causative agent for Kaposi’s sarcoma [2]. Other disease associations include non-Hodgkin’s lymphoma, epithelial neoplasia, and renal cell carcinoma. There has been only one reported case of Castleman’s disease that coexisted with sarcoidosis [3]. Here, we present another case where coincidental sarcoidosis was found after the patient demonstrated a radiologic mixed response to an anti-interleukin-6 (anti-IL-6) monoclonal antibody (CNTO 328; siltuximab) that was recently approved for the treatment of HIV-negative and HHV-8-negative multicentric Castleman’s disease [4].

## Case presentation

**Figure 1:**
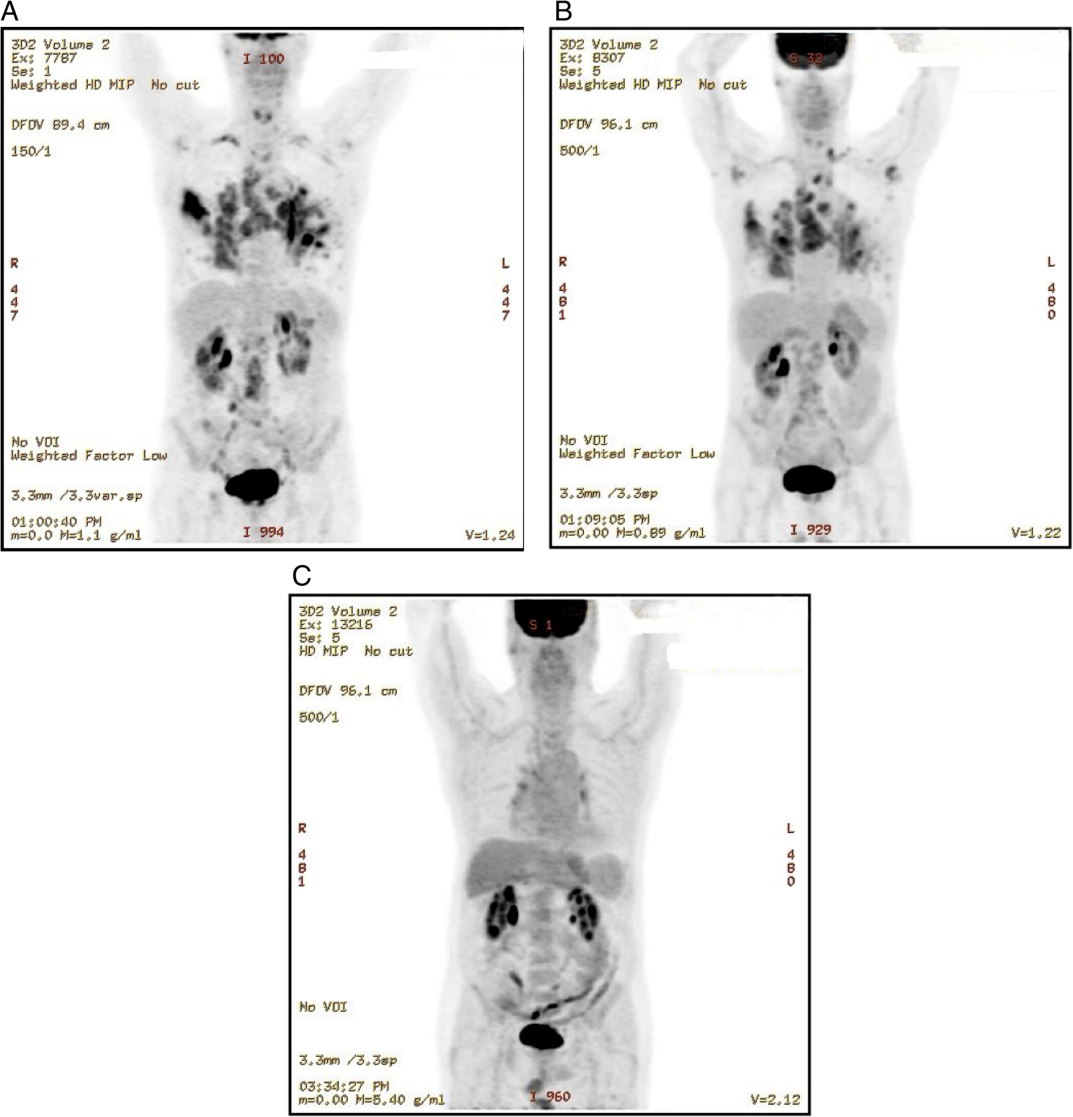
**Fluorodeoxyglucose positron emission tomography computed tomography scans. (A)** Baseline images show lung and mediastinal disease. **(B)** Images after 5 months of treatment with siltuximab show a mixed response with improvement in lungs and worsening in mediastinum. **(C)** After 3.5 years of siltuximab combined with low maintenance doses of prednisone (2.5mg orally daily).

A 76-year-old Caucasian man presented to his physician with a history of dry cough of a few months’ duration associated with fatigue, loss of appetite, and weight loss of more than 18kg (40lb). On further workup, multiple lung lesions were found on fluorodeoxyglucose positron emission tomography computed tomography (FDG-PET/CT) scan (Figure 1A), which on biopsy and pathologic review were due to Castleman’s disease. He was negative for HIV and HHV-8. He was referred to the Clinical Center for Targeted Therapy (Phase I Clinic) at MD Anderson Cancer Center for further treatment. His medical history included hypertension, hyperlipidemia, gastroesophageal reflux disease, diverticulosis, chronic obstructive pulmonary disease, and benign prostatic hypertrophy, for which he had recently undergone transurethral resection of his prostate. His family history was significant for lymphoma and prostate cancer in his brother, breast cancer in his sister, and small bowel cancer in his mother. He had last smoked cigarettes when he was in college and drank socially. He was living with his wife and had four children, all in good health. His medications included tamsulosin, simvastatin, omeprazole, and a multivitamin.

He was started on siltuximab, previously known as CNTO 328, a chimeric, human–murine anti-IL-6 antibody that binds and neutralizes IL-6 with high affinity (dose = 11mg/kg intravenously every 3 weeks). This treatment was given as part of a trial previously reported, and all procedures including consent were performed in accordance with the guidelines of the MD Anderson Cancer Center Internal Review Board [5]. He tolerated the treatment well and although he complained of intermittent fatigue, he was able to ride his bicycle up to 16km (10 miles) in a day. FDG-PET/CT scans showed significant improvement in his lungs, but worsening mediastinal disease after approximately 5 months, consistent with a mixed response (Figure 1B). In order to elucidate the reason for mixed response, a needle biopsy was performed that showed a lymphoplasmacytic infiltrate with non-caseating, ill-defined granulomas and scarring consistent with sarcoidosis. Prednisone 50mg by mouth daily was started, which was tapered to 5mg daily. This was intermittently restarted at least two more times, but he was maintained on a dose of 2.5 to 5mg daily for most of his subsequent course. Siltuximab was continued. A subsequent FDG-PET/CT scan (Figure 1C) showed near-complete resolution of lung and mediastinal disease, and he has continued to receive treatment for 3.5 years without serious adverse events. He has regained the 18kg (40lb) lost before treatment and is actively bicycling about 48 to 64km (30 to 40 miles) per week.

## Discussion

Castleman’s disease is a rare lymphoproliferative disorder that was first described as localized lymphoid hyperplasia involving mainly the mediastinal group of lymph nodes [1]. Two forms of the disease exist: a localized or unicentric form involving only a group of lymph nodes and a multicentric form that involves different groups of lymph nodes in different areas of the body. The multicentric form of Castleman’s disease recently has been found to be at times associated with HIV and HHV-8 infections. On histological examination, it is usually divided into the hyalinized vascular type and the plasma cell variant type, the latter being more common in the multicentric type of the disease. There also exists a mixed variant [6,7]. Unicentric Castleman’s disease usually presents in early adulthood with localized masses in the mediastinum, rarely causing systemic symptoms. In contrast, the multicentric form of Castleman’s disease has been found to occur in the fifth or sixth decade of life, presenting with vague constitutional symptoms such as fever, night sweats, fatigue, and weight loss. Various laboratory abnormalities have also been found, including anemia, thrombocytosis, thrombocytopenia, hypoalbuminemia, hypergammaglobulinemia, and increased C-reactive protein and erythrocyte sedimentation rate [8,9].

Lymphoproliferation in Castleman’s disease is thought to be driven by IL-6, a cytokine produced by various cells including T cells and B cells in response to various inflammatory disease processes [9-14]. It stimulates the production and differentiation of cells, including B cells. Elevated levels of IL-6 have been demonstrated in murine models of Castleman’s disease-like syndrome [9]. Various other mediators have also been identified that could be involved in the progression of the disease.

Treatment of localized forms of the disease with complete surgical excision of the affected lymph node has been found to be curative [12]. Radiation therapy is effective when surgery is not possible. However, the multicentric form of the disease requires systemic treatment. Medications that have been used include corticosteroids, antibody against CD20 (rituximab), and, more recently, there has been considerable success with antibodies targeting IL-6. Tocilizumab, an anti-IL-6 receptor antibody, has been found to be effective and is approved in Japan for treatment of Castleman’s disease [9]. It has been reported earlier that siltuximab showed a clinical benefit response in 78% of patients with Castleman’s disease in an interim analysis of a phase 1 study [5], and this molecule was subsequently approved following a registration study [4].

Our patient had the multicentric type of Castleman’s disease, for which he received siltuximab, an anti-IL-6 antibody. He had an initial remarkable response in his lung, but his mediastinal disease worsened. On further investigation, by performing a fine-needle biopsy, the mixed response was found to be due to sarcoidosis. There has been only one previously reported case of Castleman’s disease that was shown to coexist with sarcoidosis, although lymphomas have also been associated with sarcoidosis [3,13,14]. Thus, this association is very rare. Of interest, IL-6 levels in the supernatants of cultured monocytes and alveolar macrophages have been reported to be significantly higher in patients with sarcoidosis than in controls [15]. Since IL-6 drives Castleman’s disease, these observations may provide a mechanistic link between the two disorders.

## Conclusions

This case represents only the second report of coexisting Castleman’s disease and sarcoidosis. Of importance, early recognition of the presence of sarcoidosis in our patient prevented discontinuation of siltuximab therapy [4,5,16], as progression was due to the sarcoidosis, rather than Castleman’s disease. Our experience also suggests that “mixed responses” should be confirmed by performing biopsies on progressive tumors. Once sarcoidosis was recognized to be the cause of worsening of symptoms and disease on imaging, steroid therapy was started, and the patient is doing well with near-complete resolution of the disease after more than 3 years of siltuximab combined with very low-dose steroids.

## Consent

Written informed consent was obtained from the patient for publication of this Case report and any accompanying images. A copy of the written consent is available for review by the Editor-in-Chief of this journal.

## Abbreviations

FDG-PET/CT: Fluorodeoxyglucose positron emission tomography computed tomography
HHV-8: Human herpesvirus 8
HIV: Human immunodeficiency virus
IL-6: Interleukin-6
